# Return to work following traumatic fractures of the thoracolumbar spine without spinal cord injury: a scoping review

**DOI:** 10.2340/17453674.2026.45364

**Published:** 2026-02-16

**Authors:** Mathijs A M SUIJKERBUIJK, Daan CRANENBROEK, Sara I VAN AMEIJDEN, Pim W VAN EGMOND, Margot C W JOOSEN, Mariska A C DE JONGH, Ruth E GEUZE

**Affiliations:** 1 Department of Orthopaedic Surgery, Elisabeth-TweeSteden Hospital, Tilburg; 2 Network Emergency Care Brabant, Elisabeth-TweeSteden Hospital, Tilburg; 3 Department of General Surgery, Elisabeth-TweeSteden Hospital, Tilburg; 4 Tranzo Scientific Center for Care and Well-Being, Tilburg School of Social and Behavioral Sciences, Tilburg University, Tilburg, the Netherlands

## Abstract

**Background and purpose:**

Traumatic fractures of the thoracolumbar spine happen in the younger, working-age population and often compromise return to work (RTW), a key factor in functional recovery and overall quality of life. Our review summarizes the current evidence on RTW following traumatic thoracolumbar fractures without spinal cord injury.

**Methods:**

A comprehensive literature search was conducted in Embase, Medline (OvidSP), Web of Science, CINAHL and Cochrane through July 2025. Studies were included if they met the following criteria: (i) traumatic thoracolumbar spine fracture without spinal cord injury, (ii) RTW reported as an outcome measure, (iii) prospective or retrospective cohort study or case-control design, and (iv) availability of a full-text article. Risk of bias was assessed for each included study.

**Results:**

31 studies met the inclusion criteria. Follow-up ranged from 3 to 226 months. Only 8 out of 31 studies were rated as low risk of bias. Reported RTW rates varied widely: 25% to 100% after surgical treatment (n = 19 studies) and 38% to 100% after non-surgical treatment (n = 19 studies). Pooled estimates showed that the mean RTW is between 76% and 84% in patients with a thoracolumbar spine fracture, irrespective of treatment modality.

**Conclusion:**

Estimated RTW rates range between 76% and 84%.

The incidence of thoracolumbar spine fractures is rising, with an estimated rate of 25 per 100,000 annually [1]. While this trend is largely driven by a rise in osteoporotic fractures [1-3], the number of younger, working-age patients sustaining traumatic vertebral fractures is also increasing [2,3]. In this latter group, such injuries frequently result in temporary or permanent work incapacity. Consequently, return to work (RTW) is a key aspect of functional recovery and plays an important role in overall quality of life [4-6].

Beyond individual consequences, the inability to return to work has substantial socioeconomic implications, including productivity loss, increased healthcare utilization, and rising social benefit expenditures [7]. RTW outcomes have been extensively studied in elective orthopedic procedures such as total hip and knee arthroplasty, with reported RTW rates ranging from 25–95% and 71–89%, respectively. However, these interventions typically allow for prehabilitation and structured recovery planning [8,9]. Similarly, the estimated RTW rate after elective lumbar fusion is about 76% within 2 years of surgery [10]. In contrast, traumatic injuries provide little opportunity for such preparation and may lead to prolonged disability due to pain, reduced mobility, and psychological distress. Persistent symptoms, including pain, limited function, and anxiety, further compromise long-term quality of life [11].

Most patients with thoracolumbar fractures do not sustain neurological deficits [12,13]. These patients face vastly different clinical pathways than those with spinal cord injury, whose rehabilitation needs are substantially more complex and fall outside the scope of this review. Despite the clinical and socioeconomic importance of RTW in this patient group, data remains scarce and fragmented. A better understanding of RTW outcomes after thoracolumbar fractures without neurological involvement would not only enable clinicians to provide more accurate prognostic information and tailor rehabilitation strategies but also allow patients to develop realistic expectations regarding their recovery and future work capacity. Therefore, our review aims to synthesize current evidence on RTW following traumatic thoracolumbar fractures without spinal cord injury.

## Methods

### Study protocol

This scoping review was not guided by a prespecified protocol. Given the exploratory nature of the review and the anticipated heterogeneity in study designs, interventions, and RTW outcomes, eligibility criteria and data charting procedures were refined iteratively during the review process. All methodological decisions were defined prior to data synthesis and are reported transparently in accordance with the PRISMA-ScR guidelines [14].

### Search strategy

The search strategy (Supplementary Table 1) was carried out on literature from the following electronic databases: Embase, Medline (OvidSP), Web of Science, CINAHL, and Cochrane. The search covered all records from each database’s inception until July 26, 2025. Additionally, the reference lists of the included studies were reviewed for relevant sources. PubMed and Google Scholar were utilized to retrieve full-text articles that were not accessible through the previously mentioned databases. This scoping review was performed using the PRISMA-ScR checklist for scoping reviews [15].

### Eligibility criteria

Studies were considered eligible if they met the following criteria: (i) traumatic fracture of the thoracolumbar spine without spinal cord injury, (ii) return to work reported as an outcome measure, (iii) study design being a prospective study, retrospective cohort study, or case-control study, and (iv) availability of a full-text article. Studies on cervical or sacral fractures, and animal studies were excluded. The search was limited to articles published in English, German, French, or Dutch.

### Identification of eligible studies

Two reviewers (MS, SA) independently screened the identified studies based on title and abstract. Full-text versions of the selected studies were then assessed, and those meeting the eligibility criteria were included in the review. Any disagreements were resolved through consensus.

### Data charting

Two independent reviewers (MS, DC) extracted data from each study based on a calibrated form, including year of publication, study design, number of included subjects, sex, mean age, treatment modality (surgical or non-surgical), fracture type and location, mean follow-up duration following trauma, and imaging technique. The primary outcome measure was RTW. When possible, RTW was classified using the validated Denis Working Scale (Table 1). In this study, successful return to work was defined as achieving at least W3 on the Denis Working Scale.

**Table 1:** Denis Working Scale

W1	Return to previous employment (heavy labor) or physical demanding activities.
W2	Able to return to previous employment (sedentary) or return to heavy labor with lifting restrictions.
W3	Unable to return to previous employment but working full-time at a new job.
W4	Unable to return to full-time work.
W5	No work, completely disabled.

### Risk of bias assessment

Methodological quality was assessed using a 12-point checklist adapted from existing tools (Supplementary Table 2) [16-18]. Studies scoring >8 were rated high quality, 5–8 moderate, and <5 low. Each criterion met received 1 point; unmet or unreported criteria scored 0.

### Statistics

Data was systematically extracted on study characteristics, patient demographics, treatment, fracture classification, imaging, follow-up, and RTW outcomes (Table 2). Subsequently, the studies were categorized based on treatment modality and follow-up duration (< 1 year vs ≥ 1 year). We assessed the normality of the RTW rates using the Shapiro–Wilk test. For all subgroups, the test indicated no significant deviation from a normal distribution (all P > 0.05). Therefore, mean RTW rates and standard deviations (SD) were calculated for each subgroup using SPSS Statistics V.23 (IBM Corp, Armonk, NY, USA).

**Table 2:** Data extraction table

Reference	Study design country	Study participants		Treatment modality		Fracture segment	Type	Mean follow-up, months	Imaging technique
		Total n	Males, n (%)	Surgical (n)	Non-surgical (n)				
Siebenga 2006 [21]	Prospective randomized trial Netherlands	32	22 (63)	Posterior fixation (17)	Brace (15)	Thoracic, lumbar	AO Type A	52	Radiograph, CT
Reid 1988 [38]	Prospective c. Canada	21	N/A	–	Orthosis	Thoracic, lumbar	Burst	18	Radiograph, CT
Huler 1991 [42]	Prospective c. USA	39	24 (62)	Posterior fixation	–	Thoracic, lumbar	Burst	24	NS
Cantor 1993 [34]	Prospective c. USA	18	9 (50)	–	Orthosis	Thoracic, lumbar	Burst	19	Radiograph, CT
Wood 2003 [20]	Prospective c. USA	47	32 (68)	Posterior or anterior fusion (24)	Body cast or orthosis (23)	Thoracic, lumbar	Burst	44	Radiograph, CT
Leferink 2003 [24]	Prospective c. Netherlands	19	10 (53)	Posterior fixation	–	Thoracic, lumbar	Burst	NS	NS
Alanay 2004 [27]	Prospective c. Turkey	15	7 (47)	–	Reduction and cast	Thoracic, lumbar	Burst	31	CT and MRI
Schmid 2012 [22]	Prospective c. Austria	35	24 (69)	TLIF (21) vs posteroanterior fusion (14)	–	Thoracic, lumbar	Burst	20	Radiograph, CT
Cimatti 2013 [43]	Prospective c. Italy	32	16 (50)	Posterior fixation, 2 techn.	–	Thoracic, lumbar	Burst	36	Radiograph, CT
Maestretti 2014 [23]	Prospective c. Switzerland	21	13 (62)	Kyphoplasty	–	Thoracic, lumbar	Comp.	120	Radiograph, CT, MRI (if needed)
Wood 2015 [19]	Prospective c. USA	37	18 (49)	Posterior or anterior fusion (19)	Body cast or orthosis (18)	Thoracic, lumbar	Burst	216	Radiograph, CT
Wall 2017 [36]	Prospective c. USA	38	21 (55)	Kypho-/vertebro-plasty (3), ORIF (9)	NS (26)	Thoracic, lumbar	Burst	NS	Radiograph
De Gendt 2020 [25]	Prospective c. Netherlands	17	6 (35)	Posterior fusion (17) **^a^**	–	Thoracic, lumbar	Burst	166	Radiograph, CT
Denis 1984 [12]	Retrospective c. USA	52	NS	Posterior fixation (13)	Unbraced or braced (35)	Thoracic, lumbar	Burst	39	CT
Knight 1993 [40]	Retrospective c. USA	22	12 (55)	Posterior fusion (11) Anterior fusion (1)	Various orthoses (10)	Lumbar	Burst	25	Radiograph, CT
Chow 1996 [44]	Retrospective c. USA	24	10 (71)	–	Hyperextension casting/bracing	Thoracic, lumbar	Burst	34	CT
Okuyama 1996 [39]	Retrospective c. Japan	19	10 (53)	Anterior fusion	–	Thoracic, lumbar	Burst	54	Radiograph, CT
Shen 1999 [37]	Retrospective c. Taiwan	38	22 (58)	–	Brace (9) Functional (29)	Thoracic, lumbar	Burst	49	Radiograph, CT
Andress 2002 [47]	Retrospective c. Germany	50	27 (54)	Posterior fixation	–	Thoracic, lumbar	Burst	68	Radiograph, CT
Tropiano 2003 [15]	Retrospective c. USA	45	30 (67)	–	Closed reduction and casting	Thoracic, lumbar	Burst	34	Radiograph, CT
Butler 2005 [45]	Retrospective c. Ireland	26	NS	Posterior fusion (11)	Casting followed by brace (15)	Lumbar	Burst	43	Radiograph, CT
Post 2006 [29]	Retrospective c. Netherlands	33	20 (61)	–	Unbraced (15) Braced (18)	Thoracic, lumbar	Comp,	NS	NS
Butler 2007 [46]	Retrospective Ireland c.	14	12 (86)	Posterior fusion (4)	Orthosis (10)	Lumbar	Burst	71	Radiograph, CT
Ozturk 2012 [30]	Retrospective c. Turkey	26	14 (54)	–	Orthosis	Thoracic, lumbar	Burst	49	Radiograph, CT, MRI
Jaffray 2015 [41]	Retrospective c. UK	60	NS	–	Orthosis	Thoracic, lumbar	Burst	3	Radiograph, CT and/or MRI
Rava 2019 [28]	Retrospective Italy c.	74	41 (55)	–	Closed reduction + cast	Thoracic, lumbar	Wedge, split/burst	28	Radiograph, CT, MRI
La Maida 2019 [32]	Retrospective c. Italy	20	10 (50)	Posterior fixation	–	Thoracic, lumbar	Comp. **^b^**	24	Radiograph, CT and MRI
Brandicourt 2021 [35]	Retrospective c. France	30	19 (63)	Posterior fixation	–	Thoracic, lumbar	NS	174	NS
Kultur 2024 [33]	Retrospective c. Turkey	12	6 (50)	Posterior fusion	–	Thoracic, lumbar	Burst	226	Radiograph
Medici 2014 [31]	Case-control Italy	39	28 (72)	Posterior fusion (24)	Orthosis (15)	Thoracic, lumbar	Burst	6	Radiograph, CT
D’Oria 2022 [26]	Case-control Italy	102	55 (54)	Vertebro-plasty (50)	Orthosis (52)	Thoracic, lumbar	Burst	12	Radiograph

c. = cohort, Comp. = compression, CT = computed tomography, MRI = magnetic resonance imaging, ORIF = open reduction and internal fixation, NS: not specified, USA: United States of America, UK: United Kingdom, TLIF: transforaminal lumbar interbody fusion.

a + endplate reduction and cement augmentation,

b distraction, translation.

Statistical comparison of RTW rates between surgical and non-surgical treatments was not performed because of substantial heterogeneity in study design, treatment indications, follow-up duration, and intervention protocols. Consequently, a meta-analysis was not feasible.

### Data sharing plan, funding, use of AI, and disclosures

AI tools were not used. Complete disclosure of interest forms according to ICMJE are available on the article page, doi: 10.2340/17453674.2026.45364

## Results

### Literature search

**Figure 1:**
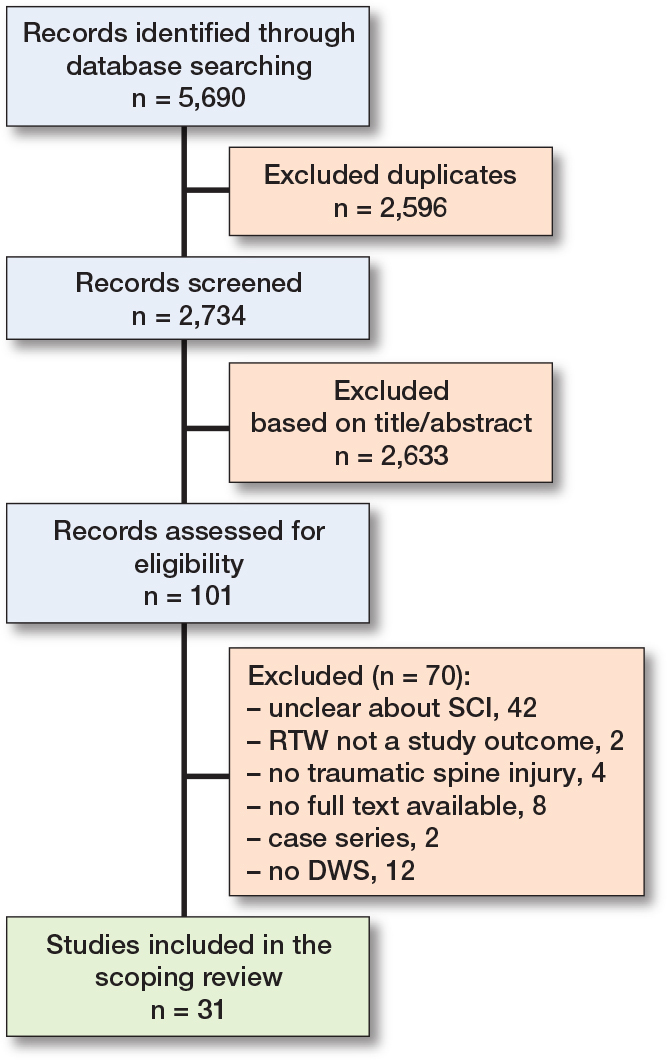
Flowchart of the included studies. DWS = Denis Work Scale; RTW = return to work; SCI = spinal cord injury.

Of the 2,734 articles identified, 2,633 were excluded based on title/abstract and 70 did not meet the inclusion criteria after full text review. Consequently, a total of 31 studies were included in the current review (Figure 1).

### Risk-of-bias assessment

Based on the predefined criteria, 8 studies were rated low risk of bias [19-27], 10 studies to have a moderate risk of bias [12,15,28-35] and 13 studies to have a high risk of bias [27,36-47] (Supplementary Table 3).

### Study characteristics

The study sizes ranged from 12 to 102 patients, with male participation between 35% and 86%. Follow-up ranged from 3 to 226 months. Surgical treatment was reported in 21 studies, non-surgical in 20 and 14 studies reported outcome after both. The included studies were performed in 13 unique countries with varying treatment practices, as well as healthcare systems. Table 2 shows the data extraction of the included studies.

### Measuring methods

24 studies used a classification system to categorize the vertebral fractures. The fracture classification systems included the Denis [48], Margerl [49], Thoracolumbar Injury Severity Score [50], or the AO Spine Thoracolumbar Injury Classification System [51]. Radiographs were most used (n = 18) for imaging assessment, often combined with CT (n = 17) and occasionally MRI (n = 5); 3 studies did not specify imaging technique. 12 studies directly used the Denis Work Scale to report RTW [12,15,23,25,26,31-34,37-39]. The remaining 19 studies did not use a specific scoring system for RTW reporting, but the Denis Work Scale could be deduced from the results [19-22,24,27-30,35,36,40-47] (Table 3).

**Table 3:** Fracture classification and scoring system to report return to work

Author, year	Fracture classification	Return to work scoring system
Siebenga 2006 [21]	AO	Not specified, but derivable **^a^**
Reid 1988 [38]	Denis	Denis Working Scale
Huler 1991 [42]	Not specified	Not specified, but derivable **^a^**
Cantor 1993 [34]	Denis	Denis Working Scale
Wood 2003 [20]	Not specified	Not specified, but derivable **^a^**
Leferink 2003 [24]	Magerl	Not specified, but derivable **^a^**
Alanay 2004 [27]	Denis	Not specified, but derivable **^a^**
Schmid 2012 [22]	Magerl	Not specified, but derivable **^a^**
Cimatti 2013 [43]	Magerl	Not specified, but derivable **^a^**
Maestretti 2014 [23]	Magerl	Denis Working Scale
Wood 2015 [19]	Not specified	Not specified, but derivable **^a^**
Wall 2017 [36]	Not specified	Not specified, but derivable **^a^**
De Gendt 2020 [25]	AO and Magerl	Denis Working Scale
Denis 1984 [12]	Denis	Denis Working Scale
Knight 1993 [40]	Denis	Not specified
Chow 1996 [44]	Denis	Not specified
Okuyama 1996 [39]	Denis	Denis Working Scale
Shen 1999 [37]	Not specified	Denis Working Scale
Andress 2002 [47]	Magerl	Not specified
Tropiano 2003 [15]	Denis	Denis Working Scale
Butler 2005 [45]	Not specified	Not specified, but derivable **^a^**
Post 2006 [29]	Magerl	Not specified, but derivable **^a^**
Butler 2007 [46]	Not specified	Not specified, but derivable **^a^**
Ozturk 2012 [30]	Denis	Denis Score for pain and function
Jaffray 2015 [41]	TLISS**^b^**	Not specified
Rava 2019 [28]	AO	Not specified, but derivable **^a^**
La Maida 2019 [32]	AO	Denis Working Scale
Brandicourt 2021 [35]	AO	Not specified, but derivable **^a^**
Kultur 2024 [33]	Denis	Denis Working Scale
Medici 2014 [31]	Magerl	Denis Working Scale
D’Oria 2022 [26]	Magerl	Denis Working Scale

a Derivable to Denis Working Scale;

b TLISS, Thoracolumbar Injury Severity Score.

### Return to work

**Figure 2:**
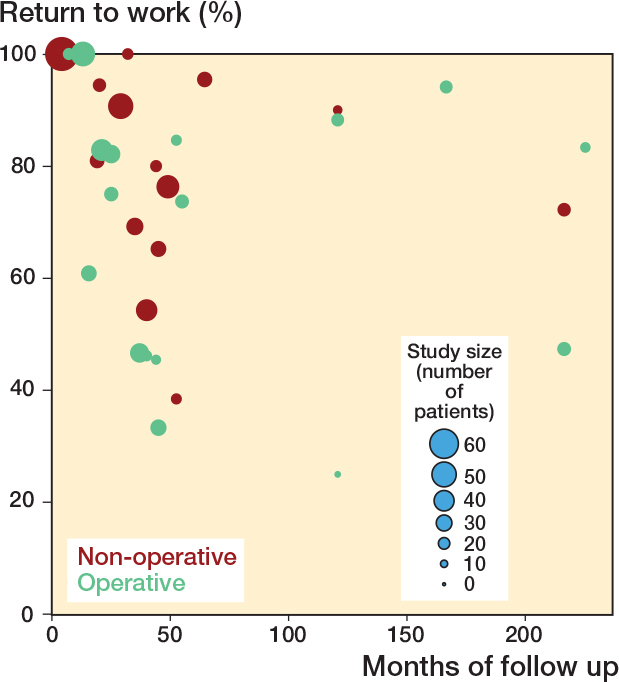
Overview of the return to work rate as reported per included study. Every dot represents one of the included studies.

Follow-up periods of the included studies ranged from 3 to 226 months. RTW rates varied widely in 19 studies on surgical treatment from 25% to 100%. Among 19 studies examining non-surgical treatment, RTW rates varied between 38% and 100%. Figure 2 demonstrates this wide variability with both surgical and non-surgical studies reporting RTW rates above 70%. Larger studies consistently clustered around 80–100%, while smaller studies showed greater heterogeneity. Pooled estimates show that the mean RTW is between 76% and 84% in patients with a thoracolumbar spine fracture, irrespective of treatment modality (Table 4).  

**Table 4:** Return to work (RTW) per treatment modality and follow-up period

Factor	< 1 year follow-up		≥ 1 year follow-up	
	n	mean RTW (SD) %	n	mean RTW (SD) %
Surgical treatment	2	100	15	64 (22)
Non-surgical treatment	4	78 (21)	14	79 (18)
Irrespective of treatment	4	84 (16)	24	76 (16)

## Discussion

This is the first review to provide an overview of RTW rates following a traumatic thoracolumbar fracture without spinal cord injury. The mean RTW rate after injury is estimated to be between 76% and 84% during follow-up spanning from 3 to 226 months.

Evidence from the broader trauma literature suggests that RTW after injury is influenced by a combination of physical limitations, psychological problems, social factors, and demographic factors. A systematic review on major trauma identified moderate evidence for the influence of age, educational level, and intensive care unit stay, with hospital length of stay emerging as one of the most consistent predictors of vocational recovery [52].

Similarly, a systematic review of biopsychosocial prognostic factors after acute orthopedic trauma demonstrated that greater injury severity is consistently associated with delayed RTW, while psychosocial aspects such as low recovery expectations, fear of reinjury, and maladaptive coping strategies are important predictors of prolonged work disability [53].

In contrast, none of the studies included in this review reported prognostic factors for RTW after thoracolumbar fractures, highlighting a critical knowledge gap. Patients understandably want to know not only if, but also when, they are likely to return to work. For many, not being able to RTW has direct financial consequences as an impact on both their well-being and quality of life. Therefore, providing prognostic information should be an essential part of patient counseling. Yet, with the current evidence, offering reliable guidance is nearly impossible. This underscores the need for future research that systematically evaluates not only physical injury characteristics but also psychosocial and sociodemographic predictors in this patient group. Such insights could ultimately inform more individualized (hospital-based) rehabilitation strategies and improve vocational outcomes.

In our review, overall RTW rates appeared broadly comparable between the 2 treatment modalities. However, heterogeneity in fracture type, treatment indication, and follow-up precludes direct comparison. Selection bias is likely, as patients with more severe or unstable fractures were generally managed surgically. Recent prospective multicenter studies provide additional insights. The international AOSpine cohort reported faster RTW after surgical management of thoracolumbar burst fractures without neurological deficit [54], and a subsequent cost-utility analysis suggested surgery may be cost-effective over a 2-year horizon compared with non-surgical care [55]. Nonetheless, current evidence remains insufficient to resolve the ongoing debate, highlighting the need for further high-quality prospective studies with standardized outcome reporting to define the optimal treatment pathway for this patient group. Future studies should explicitly address RTW expectations to better support patients in planning their rehabilitation and financial recovery.

Finally, it is important to recognize that RTW outcomes are shaped not only by clinical care but also by difference in societal and healthcare between various countries. Across the 31 included studies conducted in 13 countries, RTW rates varied widely within and across settings. Given the heterogeneity in case mix, indications for surgery, RTW definitions, and follow-up durations, we did not identify a consistent country-level pattern, and any apparent differences are likely explained by study-level factors rather than national context per se. Additionally, access to occupational healthcare and compensating welfare systems play a crucial role; early involvement of an occupational therapist or financial support after trauma have been associated with a faster RTW [35,56]. So, system-level elements (e.g., compensation schemes and access to occupational health services) may influence vocational recovery but were rarely reported and precluded formal comparison in the current study.

### Strengths

First, this is the first study to systematically synthesize the available evidence on RTW following traumatic thoracolumbar fractures without spinal cord injury. This is a clinically and socioeconomically relevant outcome that has previously received limited attention. Second, the comprehensive search strategy across multiple major databases, combined with adherence to the PRISMA-ScR guidelines, ensures a thorough and reproducible selection process. Third, a detailed risk of bias assessment was conducted using a predefined quality appraisal tool, allowing for critical evaluation of the methodological rigor of the included studies.

### Limitations

First, there was substantial heterogeneity among the included studies. The studies span a period of 32 years, during which methodological standards and clinical practices have evolved considerably. This is reflected in the risk-of-bias assessment, which identified only 7 out of the included 31 studies as having a low risk of bias. Treatment techniques also changed considerably over time, with repositioning followed by immobilization in a cast largely replaced by bracing or orthotic management, and percutaneous stabilization becoming more common than open procedures. Another considerable heterogeneity was found in the imaging modalities and fracture classification systems, which introduces a substantial risk of misclassification and may have influenced the reported RTW rates.

Although a statistical comparison between surgical and non-surgical groups would have been feasible, this was not performed due to the pronounced heterogeneity in study design, treatment indications, and follow-up. Notably, both surgical and non-surgical treatment protocols varied considerably, and indications for specific interventions were often not clearly defined.

The variation in RTW outcomes and follow-up is visually demonstrated in Figure 2, which presents RTW percentages from individual studies, stratified by treatment modality. To enable objective and consistent comparison across studies, the Denis Work Scale was employed to estimate RTW rates. Nonetheless, it remains unclear how “full-time work” was defined across studies and whether patients had a preference or history of full-time employment prior to injury, further limiting interpretation of reported RTW outcomes.

### Conclusions

Our review shows that the RTW rate following thoracolumbar spine fractures without spinal cord injury is estimated to be between 76% and 84%, irrespective of treatment modality. However, the considerable heterogeneity across studies in various aspects prevents firm conclusions about the actual RTW rate and underscores the urgent need for high-quality research on clinical outcomes in this patient population. Furthermore, the current evidence does not allow identification of factors predicting delayed or failed RTW.

*In perspective*, it is crucial to include RTW outcomes in future standardized and prospective research to address these gaps effectively. Only then can we truly guide our patients with the clarity they need, clarity that is essential not only for recovery but also for safeguarding their financial security and quality of life after trauma.

### Supplementary data

Supplementary Tables 1–3 are available as Supplementary data on the article page, doi: 10.2340/17453674.2026.45364

## Supplementary Material



## Notes

MS: conceptualization, methodology, data collection and verification, formal analysis, writing (original draft), visualization. DC: data collection, data curation, formal analysis. SA: data collection and verification, formal analysis. PvE: conceptualization, methodology, validation, visualization, writing (review). MJ: writing (review). MdJ: methodology, writing (review). RG: conceptualization, methodology, writing (review) and visualization.

The authors are grateful to W. Bramer, medical librarian of the Erasmus MC, for his assistance in performing the literature search.  

Handling co-editors: Paul Gerdhem and Robin Christensen

*Acta* thanks Simon Blixt and Lukas Bobinski for help with peer review of this manuscript.

## References

[1] den Ouden L P, Smits A. J, Stadhouder A, Feller R, Deunk J, Bloemers FW. Epidemiology of spinal fractures in a level one trauma center in the Netherlands: A 10 YEARS REVIEW. Spine (Phila Pa 1976) 2019; 44: 732-9. doi: 10.1097/BRS.0000000000002923. 30395086

[2] Bruggink C, van de Ree C L P, van Ditshuizen J, Polinder-Bos H A, Oner F C, Reijman M, et al. Increased incidence of traumatic spinal injury in patients aged 65 years and older in the Netherlands. Eur Spine J 2024; 33: 3677-84. doi: 10.1007/s00586-024-08310-w. 38836903

[3] Blecher R, Yilmaz E, Ishak B, von Glinski A, Moisi M, Oskouian R. J, et al. Uptrend of cervical and sacral fractures underlie increase in spinal fractures in the elderly, 2003–2017: analysis of a state-wide population database. Eur Spine J 2020; 29: 2543-9. doi: 10.1007/s00586-020-06553-0. 32577864

[4] Chan S K, Man D W. Barriers to returning to work for people with spinal cord injuries: a focus group study. Work 2005; 25: 325-32. doi: 10.3233/WOR-2005-25406. 16340109

[5] Murphy G C, Middleton J, Quirk R, De Wolf A, Cameron I D. Predicting employment status at 2 years’ postdischarge from spinal cord injury rehabilitation. Rehabil Psychol 2011; 56: 251-6. doi: 10.1037/a0024787. 21787095

[6] van Ditshuizen J C, van Lieshout E M M, van Beeck E F, Verhofstad M H J, den Hartog D, Dutch Trauma Registry S. Health-related quality of life and return to work 1 year after major trauma from a network perspective. Eur J Trauma Emerg Surg 2022; 48: 242131. doi: 10.1007/s00068-021-01736-9. PMC9192406 34514511

[7] de Munter L, Geraerds A, de Jongh M A C, van der Vlegel M, Steyerberg E W, Haagsma J A, et al. Prognostic factors for medical and productivity costs, and return to work after trauma. PLoS One 2020; 15: e0230641. doi: 10.1371/journal.pone.0230641. PMC7094860 32210472

[8] Tilbury C, Schaasberg W, Plevier J W, Fiocco M, Nelissen R G, Vliet Vlieland T P. Return to work after total hip and knee arthroplasty: a systematic review. Rheumatology (Oxford) 2014; 53: 512-25. doi: 10.1093/rheumatology/ket387. 24273048

[9] Van Leemput D, Neirynck J, Berger P, Vandenneucker H. Return to work after primary total knee arthroplasty under the age of 65 years: a systematic review. J Knee Surg 2022; 35: 1249-59. doi: 10.1055/s-0041-1742090. 33472262

[10] Laurén J L C, Toivonen L A, Repo J P, Kautiainen H, Häkkinen A H, Neva M H. Return to work within 2 years of lumbar fusion: a prospective cohort study. Acta Orthop 2025; 96: 612-17. doi: 10.2340/17453674.2025.43751. 40814980 PMC12357179

[11] Zidén L, Scherman M H, Wenestam C G. The break remains – elderly people’s experiences of a hip fracture 1 year after discharge. Disabil Rehabil 2010; 32: 103-13. doi: 10.09/09638280903005784. 19562584

[12] Denis F, Armstrong G W, Searls K, Matta L. Acute thoracolumbar burst fractures in the absence of neurologic deficit: a comparison between operative and nonoperative treatment. Clin Orthop Relat Res 1984: 142-9. doi: 10.1097/00003086-198406000-00019. 6478691

[13] Mukherjee S, Beck C, Yoganandan N, Rao R D. Incidence and mechanism of neurological deficit after thoracolumbar fractures sustained in motor vehicle collisions. J Neurosurg Spine 2016; 24: 323-31. doi: 10.3171/2015.8.SPINE15418. 26451664

[14] Tricco A C, Lillie E, Zarin W, O’Brien K K, Colquhoun H, Levac D, et al. PRISMA Extension for Scoping Reviews (PRISMA-ScR): checklist and explanation. Ann Intern Med 2018; 169: 467-73. doi: 10.7326/M18-0850. 30178033

[15] Tropiano P, Huang R C, Louis C A, Poitout D G, Louis R P. Functional and radiographic outcome of thoracolumbar and lumbar burst fractures managed by closed orthopaedic reduction and casting. Spine (Phila Pa 1976) 2003; 28: 2459-65. doi: 10.1097/01.BRS.0000093497.29680.19. 14595164

[16] Deeks J J, Dinnes J, D’Amico R, Sowden A J, Sakarovitch C, Song F, et al. Evaluating non-randomised intervention studies. Health Technol Assess 2003; 7: iii-x, 1-173. doi: 10.3310/hta7270. 14499048

[17] Downs S H, Black N. The feasibility of creating a checklist for the assessment of the methodological quality both of randomised and non-randomised studies of health care interventions. J Epidemiol Community Health 1998; 52: 37784. doi: 10.1136/jech.52.6.377. PMC1756728 9764259

[18] Slim K, Nini E, Forestier D, Kwiatkowski F, Panis Y, Chipponi J. Methodological index for non-randomized studies (minors): development and validation of a new instrument. ANZ J Surg 2003; 73: 712-16. doi: 10.1046/j.1445-2197.2003.02748.x. 12956787

[19] Wood K B, Buttermann G R, Phukan R, Harrod C C, Mehbod A, Shannon B, et al. Operative compared with nonoperative treatment of a thoracolumbar burst fracture without neurological deficit: a prospective randomized study with follow-up at sixteen to twenty-two years. J Bone Joint Surg Am 2015; 97: 3-9. 25568388 10.2106/JBJS.N.00226

[20] Wood K, Buttermann G, Mehbod A, Garvey T, Jhanjee R, Sechriest V. Operative compared with nonoperative treatment of a thoracolumbar burst fracture without neurological deficit: a prospective, randomized study. J Bone Joint Surg Am 2003; 85: 773-81. doi: 10.2106/JBJS.N.00851. 12728024

[21] Siebenga J, Leferink V J, Segers M J, Elzinga M J, Bakker F C, Haarman H J, et al. Treatment of traumatic thoracolumbar spine fractures: a multicenter prospective randomized study of operative versus nonsurgical treatment. Spine (Phila Pa 1976) 2006; 31: 2881-90. doi: 10.1097/01.brs.0000247804.91869.1e 17139218

[22] Schmid R, Lindtner R A, Lill M, Blauth M, Krappinger D, Kammerlander C. Combined posteroanterior fusion versus transforaminal lumbar interbody fusion (TLIF) in thoracolumbar burst fractures. Injury 2012; 43: 475-9. doi: 10.1016/j.injury.2011.12.011. 22227107

[23] Maestretti G, Sutter P, Monnard E, Ciarpaglini R, Wahl P, Hoogewoud H, et al. A prospective study of percutaneous balloon kyphoplasty with calcium phosphate cement in traumatic vertebral fractures: 10-year results. Eur Spine J 2014; 23: 1354-60. doi: 10.1007/s00586-014-3206-1. 24509773

[24] Leferink V J, Keizer H J, Oosterhuis J K, van der Sluis C K, ten Duis H J. Functional outcome in patients with thoracolumbar burst fractures treated with dorsal instrumentation and transpedicular cancellous bone grafting. Eur Spine J 2003; 12: 261-7. doi: 10.1007/s00586-002-0513-5. 12800001 PMC3615499

[25] De Gendt E E A, Kuperus J S, Foppen W, Oner F C, Verlaan J J. Clinical, radiological, and patient-reported outcomes 13 years after pedicle screw fixation with balloon-assisted endplate reduction and cement injection. Eur Spine J 2020; 29: 914-21. doi: 10.1007/s00586-019-06261-0. 32036427

[26] D’Oria S, Dibenedetto M, Squillante E, Somma C, Hannan C. J, Giraldi D, et al. Traumatic compression fractures in thoracic-lumbar junction: vertebroplasty vs conservative management in a prospective controlled trial. J Neurointerv Surg 2022; 14: 202-6. doi: 10.1136/neurintsurg-2021-018063. 33758067

[27] Alanay A, Yazici M, Acaroglu E, Turhan E, Cila A, Surat A. Course of nonsurgical management of burst fractures with intact posterior ligamentous complex: an MRI study. Spine (Phila Pa 1976) 2004; 29: 2425-2431. doi: 10.1097/01.BRS.0000141907.76462.5C 15507806

[28] Rava A, Fusini F, Cinnella P, Massè A, Girardo M. Is cast an option in the treatment of thoracolumbar vertebral fractures? J Craniovertebr Junction Spine 2019; 10: 51-6. doi: 10.4103/jcvjs.JCVJS_21_18. 31000982 PMC6469317

[29] Post R B, Keizer H J, Leferink V J, van der Sluis C K. Functional outcome 5 years after non-operative treatment of type A spinal fractures. Eur Spine J 2006; 15: 472-8. doi: 10.1007/s00586-005-1022-7. 15937675 PMC3489326

[30] Öztürk I, Ertürer E, Sönmez M M, Sarı S, Şeker A, Seçkin M F. Early mobilization with customized TLSO brace in thoracolumbar burst fractures. Acta Orthop Traumatol Turc 2012; 46: 373-8. doi: 10.3944/AOTT.2012.2563 23268823

[31] Medici A, Meccariello L, Falzarano G. Non-operative vs. percutaneous stabilization in Magerl’s A1 or A2 thoracolumbar spine fracture in adults: is it really advantageous for a good alignment of the spine? Preliminary data from a prospective study. Eur Spine J 2014; 23(Suppl 6): 677-83. doi: 10.1007/s00586-014-3559-9 25212447

[32] La Maida G A, Ruosi C, Misaggi B. Indications for the monosegmental stabilization of thoraco-lumbar spine fractures. Int Orthop 2019; 43: 169-76. doi: 10.1007/s00264-018-4060-7. 30430192

[33] Kultur Y, Sarikaya İ, Ozsahin M K, Davulcu C D, Aydingoz O. Twenty year outcomes following short-segment posterior instrumentation and fusion for thoracolumbar burst fractures: a retrospective observational study. Medicine (Baltimore) 2024; 103: e40579. doi: 10.1097/MD.0000000000040579. 39560536 PMC11575997

[34] Cantor J B, Lebwohl N H, Garvey T, Eismont F J. Nonoperative management of stable thoracolumbar burst fractures with early ambulation and bracing. Spine (Phila Pa 1976) 1993; 18: 971-6. 8367784 10.1097/00007632-199306150-00004

[35] Brandicourt P, Luby N, Djidjeli I, Cheng I, De Barros A, Brauge D, et al. Clinical long-term consequences of thoraco-lumbar spine fracture and osteosynthesis. Orthop Traumatol Surg Res 2021; 107: 102941. doi: 10.1097/00007632-199304150-00006. 33895384

[36] Wall B A, Moskowitz A, Whitaker M C, Jones T L, Stuckey R M, Carr-Maben C L, et al. Functional outcomes of thoracolumbar junction spine fractures. Kans J Med 2017; 10: 30-4. doi: 10.17161/kjm.v10i2.8618. 29472964 PMC5733412

[37] Shen W J, Shen Y S. Nonsurgical treatment of three-column thoracolumbar junction burst fractures without neurologic deficit. Spine (Phila Pa 1976) 1999; 24: 412-15. doi: 10.1097/00007632-199902150-00017. 10065527

[38] Reid D C, Hu R, Davis L A, Saboe L A. The nonoperative treatment of burst fractures of the thoracolumbar junction. J Trauma 1988; 28: 1188-94. doi: 10.1097/00005373-198808000-00009. 3411642

[39] Okuyama K, Abe E, Chiba M, Ishikawa N, Sato K. Outcome of anterior decompression and stabilization for thoracolumbar unstable burst fractures in the absence of neurologic deficits. Spine (Phila Pa 1976) 1996; 21: 620-5. doi: 10.1097/00007632-199603010-00014. 8852319

[40] Knight R Q, Stornelli D P, Chan D. P, Devanny J R, Jackson K V. Comparison of operative versus nonoperative treatment of lumbar burst fractures. Clin Orthop Relat Res 1993: 112-21. doi: 10.1097/00003086-199308000-00015. 8339471

[41] Jaffray D C, Eisenstein S M, Balain B, Trivedi J M, Newton Ede M. Early mobilisation of thoracolumbar burst fractures without neurology: a natural history observation. Bone Joint J 2016; 98-B: 97-101. doi: 10.1302/0301-620X.98B1.36166. 26733521

[42] Huler R J, Esses S I, Botsford D J. Work status after posterior fixation of unstable but neurologically intact burst fractures of thoracolumbar spine. Paraplegia 1991; 29: 600-6. doi: 10.1038/sc.1991.108. 1787984

[43] Cimatti M, Forcato S, Polli F, Miscusi M, Frati A, Raco A. Pure percutaneous pedicle screw fixation without arthrodesis of 32 thoraco-lumbar fractures: clinical and radiological outcome with 36-month follow-up. Eur Spine J 2013; 22(Suppl 6): S925-32. doi: 10.1007/s00586-013-3000-7. 24121749 PMC3830047

[44] Chow G H, Nelson B J, Gebhard J S, Brugman J L, Brown C W, Donaldson D H. Functional outcome of thoracolumbar burst fractures managed with hyperextension casting or bracing and early mobilization. Spine (Phila Pa 1976) 1996; 21: 2170-5. doi: 10.1097/00007632-199609150-00015. 8893445

[45] Butler J S, Walsh A, O’Byrne J. Functional outcome of burst fractures of the first lumbar vertebra managed surgically and conservatively. Int Orthop 2005; 29: 51-4. doi: 10.1007/s00264-004-0612-7. 15538564 PMC3456945

[46] Butler J S, Fitzpatrick P, Ni Mhaolain A M, Synnott K, O’Byrne J M. The management and functional outcome of isolated burst fractures of the fifth lumbar vertebra. Spine (Phila Pa 1976) 2007; 32: 443-7. doi: 10.1097/BRS.0b013e31802e7e29. 17304135

[47] Andress H J, Braun H, Helmberger T, Schürmann M, Hertlein H, Hartl W H. Long-term results after posterior fixation of thoraco-lumbar burst fractures. Injury 2002; 33: 357-65. doi: 10.1016/S0020-1383(01)00147-7. 12091034

[48] Denis F. The three column spine and its significance in the classification of acute thoracolumbar spinal injuries. Spine (Phila Pa 1976) 1983; 8: 817-31. doi: 10.1097/00007632-198311000-00008. 6670016

[49] Magerl F, Aebi M, Gertzbein S D, Harms J, Nazarian S. A comprehensive classification of thoracic and lumbar injuries. Eur Spine J 1994; 3: 184-201. doi: 10.1007/BF02221591. 7866834

[50] Vaccaro A R, Lehman R A Jr, Hurlbert R J, Anderson P A, Harris M, Hedlund R, et al. A new classification of thoracolumbar injuries: the importance of injury morphology, the integrity of the posterior ligamentous complex, and neurologic status. Spine (Phila Pa 1976) 2005; 30: 2325-33. doi: 10.1097/01.brs.0000182986.43345.cb. 16227897

[51] Vaccaro A R, Oner C, Kepler C K, Dvorak M, Schnake K, Bellabarba C, et al. AOSpine thoracolumbar spine injury classification system: fracture description, neurological status, and key modifiers. Spine (Phila Pa 1976) 2013; 38: 2028-37. doi: 10.1097/BRS.0b013e3182a8a381. 23970107

[52] Neubert A, Hempe S, Bieler D, Schulz D, Jaekel C, Bernhard M, et al. Return to work after major trauma: a systematic review. Scand J Trauma Resusc Emerg Med 2025; 33: 44. doi: 10.1186/s13049-025-01144-9. 40098046 PMC11917110

[53] Duong H P, Garcia A, Hilfiker R, Léger B, Luthi F. Systematic review of biopsychosocial prognostic factors for return to work after acute orthopedic trauma: a 2020 update. Front Rehabil Sci 2021; 2: 791351. doi: 10.3389/fresc.2021.791351. 36188871 PMC9397710

[54] Dandurand C, Öner C F, Schnake K J, Bransford R J, Schroeder G D, Dea N, et al. Surgical versus nonsurgical treatment of thoracolumbar burst fractures in neurologically intact patients: a cost-utility analysis. Spine J 2025; 25: 1494-1507. doi: 10.1016/j.spinee.2025.03.012. 39892710

[55] Dvorak M F, Öner C F, Dandurand C, Schnake K J, Bransford R J, Popescu E C, et al. Surgical versus non-surgical treatment of thoracolumbar burst fractures in neurologically intact patients: a prospective international multicentre cohort study. Global Spine J 2025: 21925682251356910. doi: 10.1177/21925682251356910. PMC12226512 40605521

[56] Ghobrial G M, Jallo J. Thoracolumbar spine trauma: review of the evidence. J Neurosurg Sci 2013; 57: 115-22. doi: 10.23736/S0390-5616.13.00663-1. 23676860

