# Supplementary material for: Return to work following traumatic fractures of the thoracolumbar spine without spinal cord injury: a scoping review
Source: Acta Orthop. 2026 Feb 16;97:83–90. doi: 10.2340/17453674.2026.45364 (PMC12908126; doi:10.2340/17453674.2026.45364)
Supplement: Supplementary file 1 [file ActaO-97-45364-s1.pdf]

Supplementary Table 1. Search strings

**Medline**
Return to Work / OR Employment / OR Work Capacity Evaluation / OR (((resume* OR return* OR status* OR activit* OR back-to OR regain* OR full-time* OR part-time* OR fulltime* OR parttime* OR abilit* OR able OR inabilit* OR inable OR disab* OR capacit* OR productiv* OR reentr* OR re-entr*) ADJ10 (work* OR job OR jobs OR occupation*)) OR employ* OR unemploy* OR worker*).ab,ti,kw.) AND (Spinal Fractures/ OR ((Fracture Fixation/ OR Bone Screws/) AND Spine/) OR (((spine OR spinal* OR burst OR compression OR sacrum OR vertebr* OR thoracolumb* OR cervicothora* OR thoraco-lumb* OR cervico-thora* OR sacral* OR odontoid* OR neck OR back OR hangman* OR wedge* OR spondylodes*) ADJ6 (fracture* OR fixat* OR screw*)) OR chance-fracture*).ab,ti,kw.) NOT (*Femoral Neck Fractures / OR (femoral-neck).ti.) NOT (news OR congres* OR abstract* OR book* OR chapter* OR dissertation abstract*).pt. AND english.la
**EMBASE**
('return to work'/de OR employment/exp OR 'work disability'/de OR 'work capacity'/de OR (((resume* OR return* OR status* OR activit* OR back-to OR regain* OR full-time* OR part-time* OR fulltime* OR parttime* OR abilit* OR able OR inabilit* OR inable OR disab* OR capacit* OR productiv* OR reentr* OR re-entr*) NEAR/10 (work* OR job OR jobs OR occupation*)) OR employ* OR unemploy* OR worker*):Ab,ti,kw) AND ('spine fracture'/exp OR (('fracture fixation'/de OR 'bone screw'/de) AND spine/exp) OR ('spine fusion'/exp AND fracture/de) OR (((spine OR spinal* OR burst OR compression OR sacrum OR vertebr* OR thoracolumb* OR cervicothora* OR thoraco-lumb* OR cervico-thora* OR sacral* OR odontoid* OR neck OR back OR hangman* OR wedge* OR spondylodes*) NEAR/6 (fracture* OR fixat* OR screw*)) OR chance-fracture*):ab,ti,kw) NOT ('femoral neck fracture'/mj OR (femoral-neck):ti) NOT ([conference abstract]/lim) AND [english]/lim
**Web of science**
TS=(((((resume* OR return* OR status* OR activit* OR back-to OR regain* OR full-time* OR part-time* OR fulltime* OR parttime* OR abilit* OR able OR inabilit* OR inable OR disab* OR capacit* OR productiv* OR reentr* OR re-entr*) NEAR/10 (work* OR job OR jobs OR occupation*)) OR employ* OR unemploy* OR worker*)) AND ((((spine OR spinal* OR burst OR compression OR sacrum OR vertebr* OR thoracolumb* OR cervicothora* OR thoraco-lumb* OR cervico-thora* OR sacral* OR odontoid* OR neck OR back OR hangman* OR wedge* OR spondylodes*) NEAR/5 (fracture* OR fixat* OR screw*)) OR chance-fracture*))) NOT TI=((femoral-neck)) NOT DT=(Meeting Abstract OR Meeting Summary) AND LA=(English)
**Cochrane**
((((resume* OR return* OR status* OR activit* OR back-to OR regain* OR full-time* OR part-time* OR fulltime* OR parttime* OR abilit* OR able OR inabilit* OR inable OR disab* OR capacit* OR productiv* OR reentr* OR re-entr*) NEAR/10 (work* OR job OR jobs OR occupation*)) OR employ* OR unemploy* OR worker*):Ab,ti,kw) AND ((((spine OR spinal* OR burst OR compression OR sacrum OR vertebr* OR thoracolumb* OR cervicothora* OR thoraco-lumb* OR cervico-thora* OR sacral* OR odontoid* OR neck OR back OR hangman* OR wedge* OR spondylodes*) NEAR/6 (fracture* OR fixat* OR screw*)) OR chance-fracture*):ab,ti,kw) NOT ((femoral-neck):ti)

CINAHL
(MH Job Re-Entry OR MH Employment OR MH Work Capacity Evaluation OR TI(((resume* OR return* OR status* OR activit* OR back-to OR regain* OR full-time* OR part-time* OR fulltime* OR parttime* OR abilit* OR able OR inabilit* OR inable OR disab* OR capacit* OR productiv* OR reentr* OR re-entr*) N10 (work* OR job OR jobs OR occupation*)) OR employ* OR unemploy* OR worker*) OR AB(((resume* OR return* OR status* OR activit* OR back-to OR regain* OR full-time* OR part-time* OR fulltime* OR parttime* OR abilit* OR able OR inabilit* OR inable OR disab* OR capacit* OR productiv* OR reentr* OR re-entr*) N10 (work* OR job OR jobs OR occupation*)) OR employ* OR unemploy* OR worker*)) AND (MH Spinal Fractures OR ((MH Fracture Fixation OR MH Bone Screws) AND MH Spine) OR TI(((spine OR spinal* OR burst OR compression OR sacrum OR vertebr* OR thoracolumb* OR cervicothora* OR thoraco-lumb* OR cervico-thora* OR sacral* OR odontoid* OR neck OR back OR hangman* OR wedge* OR spondylodes*) N5 (fracture* OR fixat* OR screw*)) OR chance-fracture*) OR AB(((spine OR spinal* OR burst OR compression OR sacrum OR vertebr* OR thoracolumb* OR cervicothora* OR thoraco-lumb* OR cervico-thora* OR sacral* OR odontoid* OR neck OR back OR hangman* OR wedge* OR spondylodes*) N5 (fracture* OR fixat* OR screw*)) OR chance-fracture*)) NOT (MM Femoral Neck Fractures OR TI(femoral-neck)) AND LA(english)

Supplementary Table 2. List of criteria for risk of bias assessment

#	Question	Response
1.	Is there a clearly stated aim?	Did they have a “study question” or “main aim” or “objective”?<br><br>The question addressed should be precise and relevant in the light of the available literature<br><br>To be scored adequate, the aim of the study should be coherent with the “introduction” of the paper
2.	Inclusion of consecutive patients	Did the authors say: “consecutive patients” or “all patients during period from ... to....”
3.	A description of inclusion and exclusion criteria	Did the authors report the inclusion and exclusion criteria?
4.	Inclusion of patients	Did the authors report how many eligible patients agreed to participate (i.e. gave consent)?
5.	Prospective collection of data. Data were collected according to a protocol established before the beginning of the study.	Did they say “prospective” or “follow-up”?<br><br>The study is NOT PROSPECTIVE when the study design is a chart review or database review
6.	Was the used imaging technique to confirm thoracolumbar fracture fractures valid and reliable?	To be scored as *adequate*, at least one of the following imaging techniques should be used:<br>conventional imaging, CT or MRI<br><br>All other imaging techniques are scored as inadequate.
7.	Unbiased assessment of the study outcome and determinants	To be judged as adequate the following aspect had to be positive:<br>Outcome(s) and determinants had to be measured independently of each other.
8.	Were the determinants measures used accurate (valid and reliable)?	For studies where the determinants measures are shown to be valid and reliable, the question should be answered adequate. For studies which refer to other work of that demonstrates the determinants measures are accurate, the question should be answered as adequate.
9.	Follow-up period appropriate to the aim of the study	Did they report the follow-up period? To be judged as adequate: Minimally 3 months
10.	Loss of follow-up	To be judged as adequate the following 2 aspects had to be positive:

		Did the report the losses of follow-up?<br>Was the loss of follow-up less than 20%?
11.	Calculation of the sample size before the study was initiated	To be judged as adequate the following question had to be positive:<br>Has a calculation of the sample size be made before the study was initiated?
12.	Adequate Statistical analyses	To be judged as adequate the following aspects had to be positive:<br>There must be a description of the relationship between the determinant and the primary outcome or a comparison (with information about the statistical significance)<br>Was there adjustment for age and/or gender? If the effect of the main confounders was not investigated or confounding was demonstrated but no adjustment was made in the final analyses the question should be answered inadequate<br>Did they show variance in the reported outcome? (e.g. SD, CI)

SD: standard deviation; CI: confidence interval

Supplementary Table 3

Author, year	1	2	3	4	5	6	7	8	9	10	11	12	Risk of bias
Siebenga et al. 2006 [21]	1	1	1	1	1	1	1	1	1	1	0	1	Low
Reid et al. 1988 [38]	0	1	0	0	1	1	0	0	1	0	0	0	High
Huler et al. 1991 [42]	1	0	0	0	1	1	0	0	1	0	0	0	High
Cantor et al. 1993 [34]	1	0	1	0	1	1	0	0	1	0	0	0	Moderate
Wood et al. 2003 [20]	1	1	1	1	1	1	1	1	1	1	0	1	Low
Leferink et al. 2003 [24]	1	1	1	0	1	1	1	1	1	0	0	0	Low
Alanay et al. 2004 [27]	1	0	0	0	0	0	1	1	1	0	0	0	High
Schmid et al. 2012 [22]	1	0	1	1	1	1	1	1	1	0	0	1	Low
Cimatti et al. 2013 [43]	0	0	1	0	1	1	0	0	1	0	0	0	High
Maestretti et al. 2014 [23]	1	1	1	1	1	1	1	1	1	1	0	1	Low
Wood et al. 2015 [19]	1	1	1	1	1	1	1	1	1	1	0	1	Low
Wall et al. 2017 [36]	1	0	1	0	0	1	0	0	1	0	0	0	High
De Gendt et al. 2020 [25]	1	1	0	1	1	1	1	1	1	1	0	1	Low
Denis et al. 1984 [12]	1	0	0	0	0	1	1	1	1	0	0	0	Moderate
Knight et al. 1993 [40]	0	0	0	0	0	1	1	1	0	0	0	0	High
Chow et al. 1996 [44]	1	0	0	0	0	1	1	1	1	0	0	0	High
Okuyama et al. 1996 [39]	1	0	0	0	0	1	1	0	1	0	0	0	High
Shen et al. 1999 [37]	1	0	1	0	0	1	0	0	1	0	0	0	High
Andress et al. 2002 [47]	0	0	1	0	0	1	1	1	1	0	0	0	High
Tropiano et al. 2003 [15]	1	0	1	0	0	1	1	1	1	0	0	0	Moderate
Butler et al. 2005 [45]	0	0	1	0	0	1	1	0	1	0	0	0	High
Post et al. 2006 [29]	0	0	1	0	1	1	1	1	1	0	0	0	Moderate
Butler et al. 2007 [46]	1	0	1	0	0	1	0	0	1	0	0	0	High
Ozturk et al. 2012 [30]	1	0	1	0	0	1	1	1	1	0	0	0	Moderate
Jaffray et al. 2015 [41]	0	0	1	0	0	1	0	0	0	1	0	0	High
Rava et al. 2019 [28]	1	0	1	0	0	1	1	0	1	0	0	0	Moderate
La Maida et al. 2019 [32]	1	0	1	0	0	1	1	1	1	0	0	1	Moderate
Brandicourt et al. 2021 [35]	1	0	1	0	0	1	1	1	1	0	0	0	Moderate
Kultur et al. 2024 [33]	1	1	0	0	0	0	1	1	1	1	0	1	Moderate
Medici et al. 2014 [31]	1	1	0	0	1	1	1	0	0	0	0	0	Moderate
D’Oria et al. 2022 [26]	0	1	1	1	1	1	1	1	1	0	1	1	Low
